# Collodion Applied to Burns

**Published:** 1851-03

**Authors:** 


*Collodion applied to burns.*—Dr. Liman, of Berlin, states that he has found collodion a most excellent application to burns. He has applied it in many cases with the best results. He states that it allays the smarting, forms a protective covering, which excludes the action of the air, and is so exactly adapted to all parts that no other dressing is required. The first application is attended with some pain, but is soon followed by alleviation of the suffering, and the cure proceeds steadily without pain. Dr. Liman applied the collodion with a hair-pencil, covering the entire surface, and daily reapplying it to the fissures and uncovered parts. Dr. Liman relates one case in which it was applied in an extensive burn withi mmediate advantage, and produced a speedy cure, without there remaining contractions of the integuments.—*Ibid. from Casper's Wochenschrift.*

